# Mig-6 Inhibits Autophagy in HCC Cell Lines by Modulating miR-193a-3p

**DOI:** 10.7150/ijms.66040

**Published:** 2022-01-16

**Authors:** Lianyue Qu, Yulong Tian, Duo Hong, Fan Wang, Zixuan Li

**Affiliations:** 1 Departmentof Pharmacy, The First Affiliated Hospital of China Medical University, Shenyang, China; 2 Key Laboratory of Diagnostic Imaging and Interventional Radiology of Liaoning Province, The First Affiliated Hospital of China Medical University, Shenyang, P. R. China

**Keywords:** Mig-6, miRNAs, miR-193a-3p, TGF-β2, Apoptosis

## Abstract

Mitogen-inducible gene 6 (Mig-6) is a tumor suppressor gene that plays an important role in many types of cancers by interacting with EGFR. However, its molecular mechanism in hepatocellular carcinoma (HCC) and its relationship with miRNAs need to be elucidated. Therefore, this study aimed to explore whether Mig-6 could promote apoptosis and the inhibition of autophagy via its downstream miRNA in HCC cell lines. We used two cell lines, HepG2 and HLE, to establish Mig-6 overexpression and knockdown experiments, as well as miR-193a mimic and inhibitor experiments. The miRNA microarray profiling was also used to verify Mig-6-regulated miRNA. We found that Mig-6 induced apoptosis and reduced autophagy of HCC cell lines. miR-193a-3p is a Mig-6-regulated miRNA in the Mig-6-overexpression model. It affected the apoptosis and autophagy of HCC cells, at least partly by regulating the expression of TGF-β2. Additionally, the relationship between Mig-6 and transforming growth factor TGF-β2 was explored in depth for the first time. These findings revealed an important role of Mig-6 in the apoptosis and autophagy of HCC cells by regulating miR-193a-3p, providing a novel insight into the therapeutic target in HCC.

## Introduction

Hepatocellular carcinoma (HCC) is the most common primary liver cancer accounting for approximately 70%-90% of liver cancer cases. It ranks as the sixth most common cancer and the second most dominating reason for cancer-related deaths worldwide 1, 2. HCC is associated with high morbidity and mortality. Surgical resection is considered the most effective therapeutic method for HCC treatment. However, only a limited number of patients are suitable for potentially curative resections. Therefore, exploration of the pathogenetic mechanisms of HCC is of great importance for facilitating the improvement in patient prognosis.

The mitogen-inducible gene 6 (Mig-6) is a regulator of epidermal growth factor signaling. It is also known as a tumor suppressor induced by EGFR signaling via the RAS-MAPK pathway 3, 4. It is a cytoplasmic protein that binds to the kinase domain of EGFR and ERBB2, locking it into a catalytically inactive conformation, thus resulting in the signal attenuation of EGFR signaling^5^.The expression of Mig-6 has been demonstrated to be suppressed in liver cancer tissues, leading to increased EGFR-AKT signaling and enhanced cell migration^6^. Mig-6 has also been reported to regulate the proliferation and apoptosis of cancer cells 7, 8. The nuclear localization of Mig-6 in a recent study showed that Mig-6 was a proximal regulator of DNA damage response that promoted DNA repair 9. However, some of the regulatory effects of Mig-6 on HCC cells, such as the effect on HCC autophagy, cannot be explained by the classic EGFR pathway, forcing the exploration of new functions of Mig-6.

MicroRNAs (miRNAs) are a class of endogenous, small, noncoding RNA molecules with 19-22 single-stranded nucleotides that regulate the protein expression through the posttranscriptional regulation and degradation of transcripts 10.These noncoding RNAs regulate the gene expression by binding to the 3'-UTR of the target mRNAs and act as antisense RNAs to downregulate the expression of their target genes at the post-transcription level 11. Dysregulation of specific miRNAs can result in aberrant protein expression, leading to the critical change in various cellular processes such as proliferation, apoptosis, cell cycle, and invasion.

Many previous studies have demonstrated that miRNAs are involved in the biological behavior of HCC. For instance, miR-139-5p influences HCC cell invasion and proliferation capacities by decreasing the expression of SLITRK4 12. miRNA-206 inhibits proliferation and migration but promoted apoptosis in HCC by modulating cMET expression^13^. The downregulated expression of miR-193a-3p was studied in many types of cancers, including non-small-cell lung cancer (NSCLC)^14^, colorectal cancer^15^, and breast cancer^16^. miR-193a-3p is also associated with the proliferation, apoptosis, invasion, and migration of liver cancer cells^17^-^19^. Therefore, the underlying molecular mechanism of miR-193a-3p in HCC remains largely unknown and needs to be explored.

In this study, the enhanced expression of Mig-6 *in vitro* was found to not only impair the autophagy but also promote apoptosis of HCC cells. The miRNA expression profiling chips were adequately applied, and the potential downstream targets of miR-193a-3p were screened out using three different bioinformatics algorithms. Afterward, the effects of miR-193a-3p on the apoptosis and autophagy of HCC were analyzed. Finally, the indirect control of Mig-6 and TGF-β2 was analyzed for the first time, providing a novel insight into the Mig-6-based therapeutic approach to treat human HCC.

## Materials and Methods

### Cell culture

Human hepatocellular cancer cell lines HepG2, HLE, Hep3B, PLC/PRF/5, and HuH7 were maintained in Dulbecco's modified Eagle's medium (DMEM )medium (Biological Industries), with 10% Fetal calf serum (FBS) (Biological Industries).Cells were maintained in 5% CO2 and 37°C incubator. Cells were seeded in cell culture flask (Corning) and were passaged when the 90% density wee reached.

### Transfection of expression plasmids, RNAi, miRNA mimics, and inhibitors

The cells were seeded 24 h prior to the experiment. Empty vector (pcDNA3) or Mig-6 overexpression vector (pcDNA3-Mig-6) kindly provided by Dr Oreste Segatto were amplified by Takara Biotechnology (Dalian, China). TGF-β2 over expression vector was from Ori Gene Technologies, Mig-6 siRNA were from Gene Pharma (Shanghai, China). miR-193a mimics and inhibitors were from RIBOBIO (Guangzhou, China). Cells were seeded in 6-well plates and were transfected using Lipofectamine 3000 (Invitrogen, Carlsbad, CA, USA) at a working concentration of 100nM according to the manufacturer's protocol.

### RNA isolation and quantitative RT-PCR

Total RNA was isolated according to the manufacturer's instructions using TRIzol reagent (Invitrogen). One microgram of overall RNA was reverse-transcribed to cDNA with oligodT (Takara) and AMV reverse transcriptase (Takara). Assays to quantify miR-193a-3p were performed using TaqMan miRNA probes and TaqMan® MicroRNA Assays (Applied Biosystems, Foster City, CA, USA) according to the manufacturer's instructions. The reactions as follows:95°C for 5 min, 40 cycles of 95°C for 15s, 60°C for 1 min. Quantitative the TGF-β2 mRNA levels were performed using SoFast^TM^ EvaGreen® Supermix (Bio-Rad). The reactions as follows:95℃ for 30 s, 40 cycles of 95℃ for 5 s, 60℃ for 20 s. Light Cycler®480Ⅱ(Roche) was used to perform the real-time PCR. The primer sequences are provided below:

TGF-β2 (forward) CATCCCGCCCACTTTCTAC; TGF-β2 (reverse) TCCGTTGTTCAGGCACTCT; Actin (forward) ATAGCACAGCCTGGATAGCAACGTAC; Actin (reverse) CACCTTCTACAATGAGCTGCGTGTG.

Experiments were repeated in triplicate.

### Western blot analysis

Total proteins from cell lines were extracted using denatured in lysis buffer (CAT.78510) (Thermo Fisher Scientific), denatured in boiling water and then quantified (CAT.23226) (Thermo Fisher Scientific). Sixty micrograms of protein were separated by 10% SDS-PAGE (Bio-Rad, USA). After transferring by Trans-Blot^®^Turbo^TM^ (Bio-Rad, USA), the polyvinylidene fluoride (PVDF) membranes (Millipore, Billerica) were blocked by 5% non-fat milk, then incubated overnight at 4°C. The antibodies used were as follows: anti-Mig-6, anti-P62, anti-LC3b, anti-TGF-β2, anti-β-actin. After that, further incubation with peroxidase-coupled anti-mouse or rabbit IgG (Zhongshan jinqiao, China) at 37°C for 2 hours. An enhanced chemiluminescence (ECL) detection system (Bio-Rad, USA) was used to visualize signals. The relative density was examined using Image Lab™ Software (Bio-Rad).

### Cell apoptosis experiments

After transfection with a plasmid, miRNA mimics, or siRNA for 48 h, the cells were collected and washed twice with phosphate-buffered saline at 4°C. The cells were then resuspended in binding buffer (1×), and adjusted to 1×10^6^ cells/mL. The cells were stained using an Annexin V-FITC/PI double staining kit (Dojindo, USA) and incubated in the dark for 15 min at room temperature. Apoptosis was analyzed by flow cytometry using a BD C6 Plus flow cytometer, and the percentage of cell apoptosis was analyzed using BD Accuri^TM^ software (both from Becton-Dickinson, CA, USA).

### Evaluation of fluorescent LC3 puncta

To analyze the changes in autophagy, LC3 punctas were indicated using mRFP -GFP-LC3 adenovirus (Gene Chem, Shanghai, China). After transfection with mRFP-GFP-LC3 for 48 h, the expression of GFP and mRFP was visualized with a fluorescence microscope (Nikon). Yellow puncta represented autophagosomes, while red puncta indicated autolysosomes. Ten views were examined per slide, and the images were acquired.

### miRNA microarray profiling and analysis

Human miRNA Microarray (GeneChip miRNA 4.0, Affymetrix) was used for microRNA profiling. The quality of total RNA was evaluated on an ultraviolet spectrophotometer (NanoDrop 2000, Thermo) and by checking the integrity and purity with a nucleic acid electrophoresis analyzer (Agilent 2100 Bioanalyzer). Biotin-labeled complementary RNA (cRNA) generated by in vitro transcription reactions (Affymetrix, USA) was fragmented and hybridized with GeneChip Hybridization Oven 645 (Affymetrix, USA) at 45°C for 16 h. After hybridization, the arrays were washed; stained with a GeneChip Hybridization, Wash, and Stain Kit (Thermo Fisher Scientific); scanned using an Affymetrix GeneChip Scanner 3000 and analyzed using Transcriptome Analysis Console software. The raw data images produced from the scanner were processed into CEL files. The intensity data of each array chip were processed using the robust multi-array average, background-adjusted, and normalized. Finally, log2 transformation was performed to extract the expression values of each transcript in the probe set. The differentially expressed genes were analyzed. TargetScan, microRNA, ORG, and miRDB software were used for target gene prediction.

### Statistical analysis

Analyses were performed with SPSS version 19.0 and Origin 2019b. Student's *t-*test was used to compare the differences between the groups. All these experiments were repeated three times. *P-*values were based on the two-sided statistical analysis, and *P* <0.05 indicated a statistically significant difference. The data are presented as the mean ± SEM.

## Result

### Mig-6 induced apoptosis and reduced autophagy of HCC cell lines

**Figure 1:**
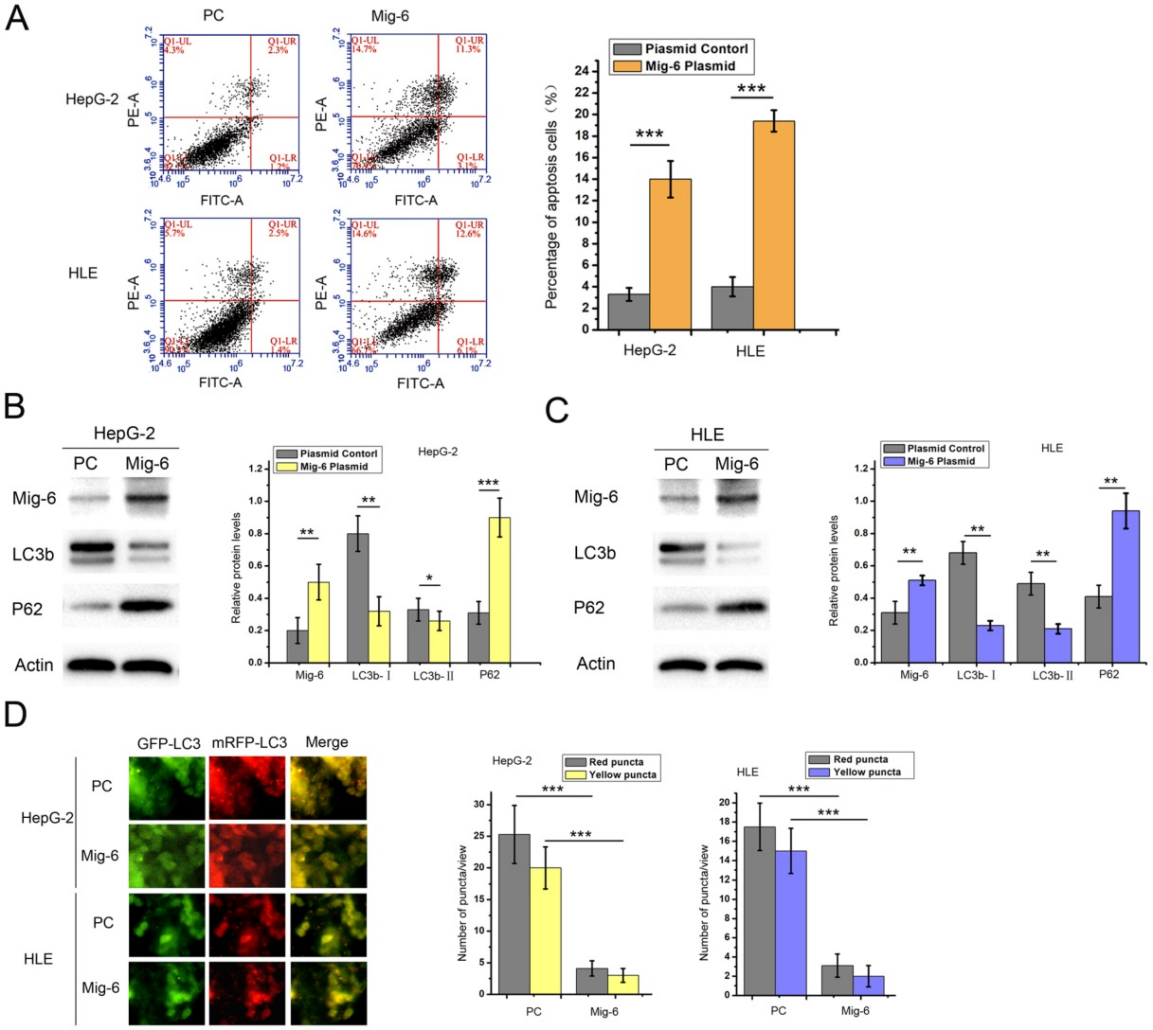
** Effect of Mig-6 plasmid on the apoptosis and autophagy of HCC cell lines.** (A) Apoptosis assay after 48-h transfection with plasmid control and Mig-6 plasmid in HepG-2 and HLE cells; the percentage of apoptosis cells was quantified.^ ***^P < 0.001. (B) Western blotting and quantitative analysis of Mig-6, LC3b, and p62 protein levels in HepG-2 cells.^ *^P < 0.05; ^**^P < 0.01; ^**^P < 0.01; ^***^P < 0.001.(C) Western blotting and quantitative analysis of Mig-6, LC3b, and p62 protein levels in HLE cells.^ **^P < 0.01. (D) Each group was transfected with a tandem mRFP-GFP-LC3 adenovirus for 24 h. Autophagosomes and autolysosomes were, respectively, visualized as yellow- and red-only punctas under a fluorescence microscope. ^***^P < 0.001.

We examined the impact of Mig-6 on HCC cell apoptosis and autophagy to validate the effect of Mig-6 in HCC cell lines. HepG-2 and HLE cells were transfected with the Mig-6 plasmid. Annexin V/PI double staining flow cytometry was applied. In the scatter plot generated using a double-variable flow cytometer, the cells in the Q1LR (Annexin V+/PI-) and Q1UR (Annexin V+/PI+) quadrants were recognized as apoptotic cells. As expected, after transfection with Mig-6, two cell lines showed an enhanced percentage of apoptosis (Figure. 1A). Meanwhile, western blot analysis was used for evaluating the expression levels of autophagy-associated proteins (Figure.1B and1C). Following transfection with Mig-6 plasmid, the expression levels of microtubule-associated protein 1 light chain 3β (LC3b) significantly decreased, while p62, which was mainly degraded by the autophagy pathway, was greatly induced compared with those in the PC groups. LC3 is the first mammalian protein found to be localized in the autophagosome membrane 20. It is localized on autophagosomes and autolysosomes. Many studies regarded LC3-II accumulation as a marker of autophagy 21. Both types of cells were transfected with mRFP-GFP-LC3 adenovirus in this study to directly visualize the changes in autophagy. The results showed that the Mig-6 plasmid significantly decreased total autophagosomes and autolysosomes, as indicated by yellow-red puncta and red puncta, respectively, in two cell lines (Figure 1D).

### Identification of Mig-6-regulated miRNAs

**Figure 2:**
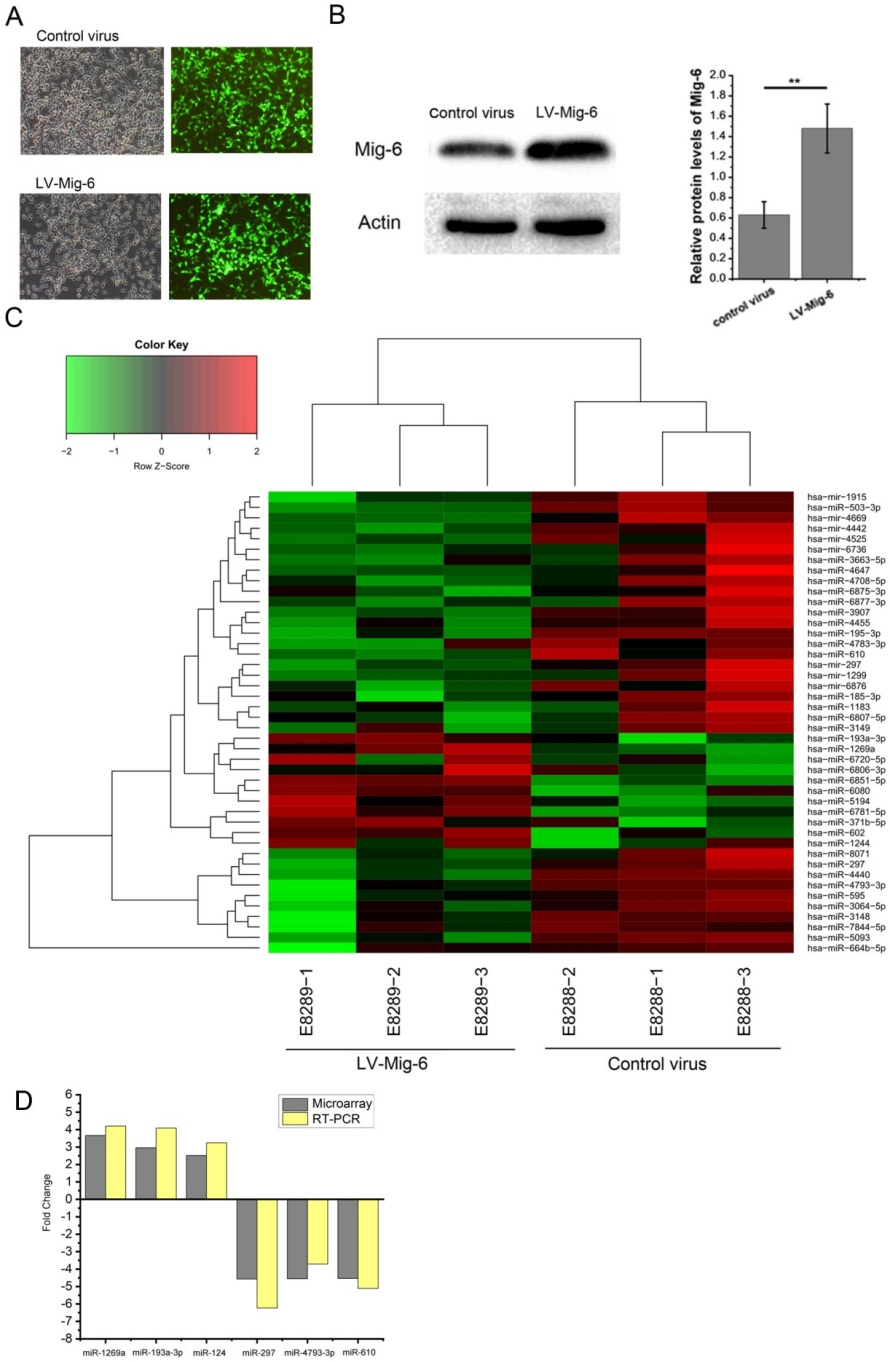
** Prediction and confirmation of Mig-6 regulated miRNAs.** (A) Visualization of GFP expression in the control virus and LV-Mig-6group (100×) after 72 h. (B) Western blotting and quantitative analysis of transduction efficiency of LV-Mig-6 in 293T cell lines. ^**^P < 0.01. (C) Hierarchical cluster analysis of Mig-6-regulated miRNAs. The horizontal axis shows comparison groups of the expression change of miRNAs. The left vertical axis shows clusters of Mig-6-regulated miRNAs whose fold change is listed in the right-hand table. Red indicates upregulated and green downregulated miRNAs. (D) Expression of some important miRNAs with more than 2.5-fold change was detected by RT-PCR using the SYBR Green method to confirm the results of the microarray.

Although several underlying mechanisms of Mig-6 were hypothesized, the regulation of miRNAs by Mig-6 was an area that had never been studied. Mig-6-regulated miRNAs were identified by transducing 293T with lentiviruses encoding control virus or lentivirus-mediated Mig-6 (Gene Chem, Shanghai, China). After 48-h transduction, the cells were treated with puromycin for 48 h to remove the nontransduced cells. Transfected cells with GFP expression were sorted for the next step (Figure 2A) which was further confirmed by western blot analysis (Figure 2B). The cells successfully expressing Mig-6 were used immediately for analysis using the GeneChip assay. The results showed that 44 Mig-6-regulated miRNAs were identified, of which 11 were upregulated and 33 downregulated (Figure 2C). Among these, the expression of 4 and 16 miRNAs in Mig-6 overexpressing cells increased or was inhibited by more than 2.5-fold, respectively (Table 1). The transcriptional changes in six miRNAs, including miR-193a-3p, were validated by the RT- PCR assay in 293T cells (Figure 2D); the related primers were purchased from Ribo Bio (Guangzhou, China) (Table S1).

**Figure 3:**
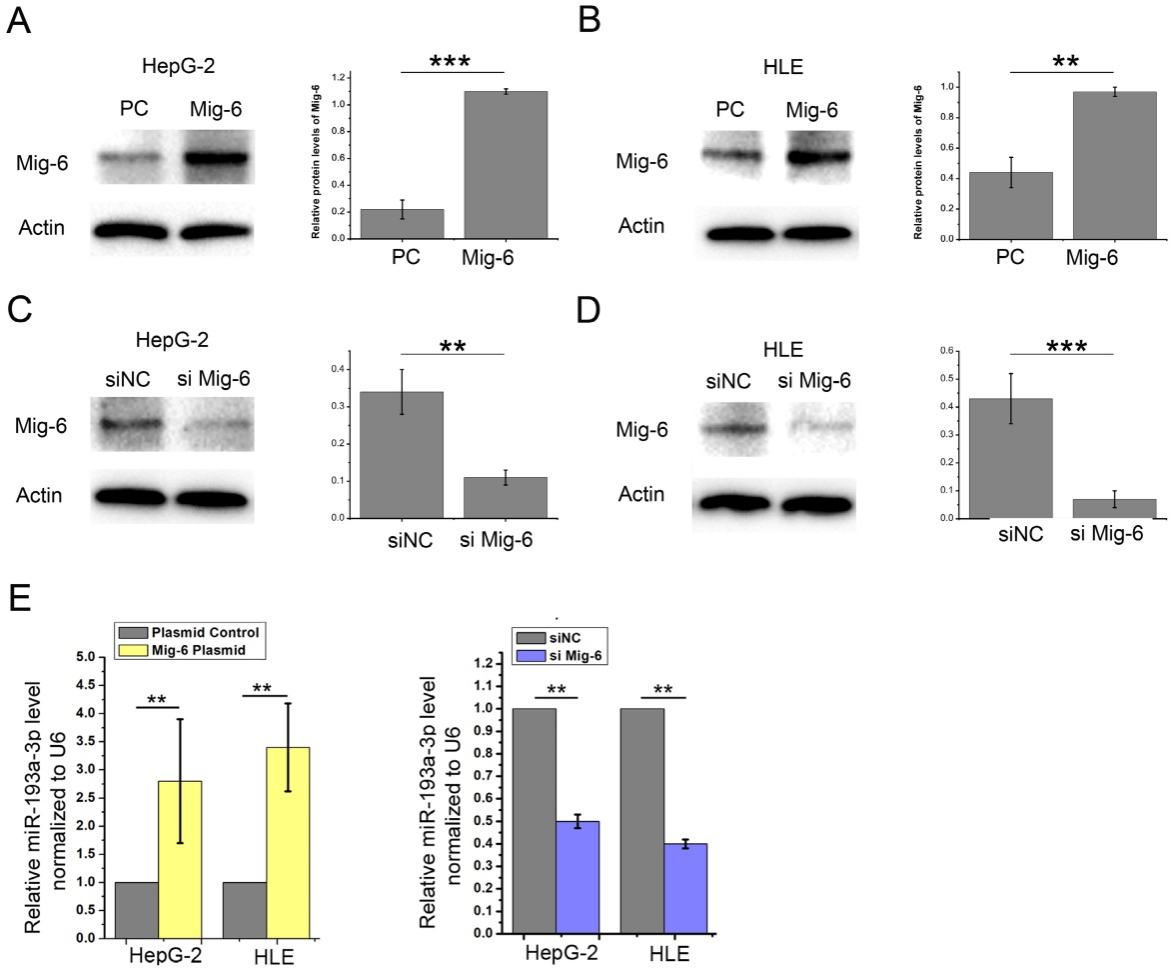
** Confirmation of Mig-6 regulated miRNAs.** (A-D) Western blotting and quantitative analysis of transfection efficiency of Mig-6 plasmid and siMig-6 in HepG-2 and HLE cell lines. ^**^P < 0.01; ^***^P < 0.001.(E) Quantitative RT-PCR analysis of the expression levels of miR-193a-3p in Mig-6 upregulated and downregulated groups. ^**^P < 0.01.

Among these miRNAs, miR-193a-3p aroused our attention for its downregulated expression in many types of cancers. The HepG-2 and HLE cells were transfected with Mig-6 plasmid and siRNA to further verify the relationship between Mig-6 and miR-193a-3p. Western blot analysis was used for detecting the transfection efficiency (Figure 3A-D). We found that the miR-193a-3p level correspondingly increased significantly after the overexpression of Mig-6; when the expression of Mig-6 was suppressed, the decrease in the miR-193a-3p level was observed (Figure 3E).

### miR-193a-3p inhibited autophagy of HCC cell lines

**Figure 4:**
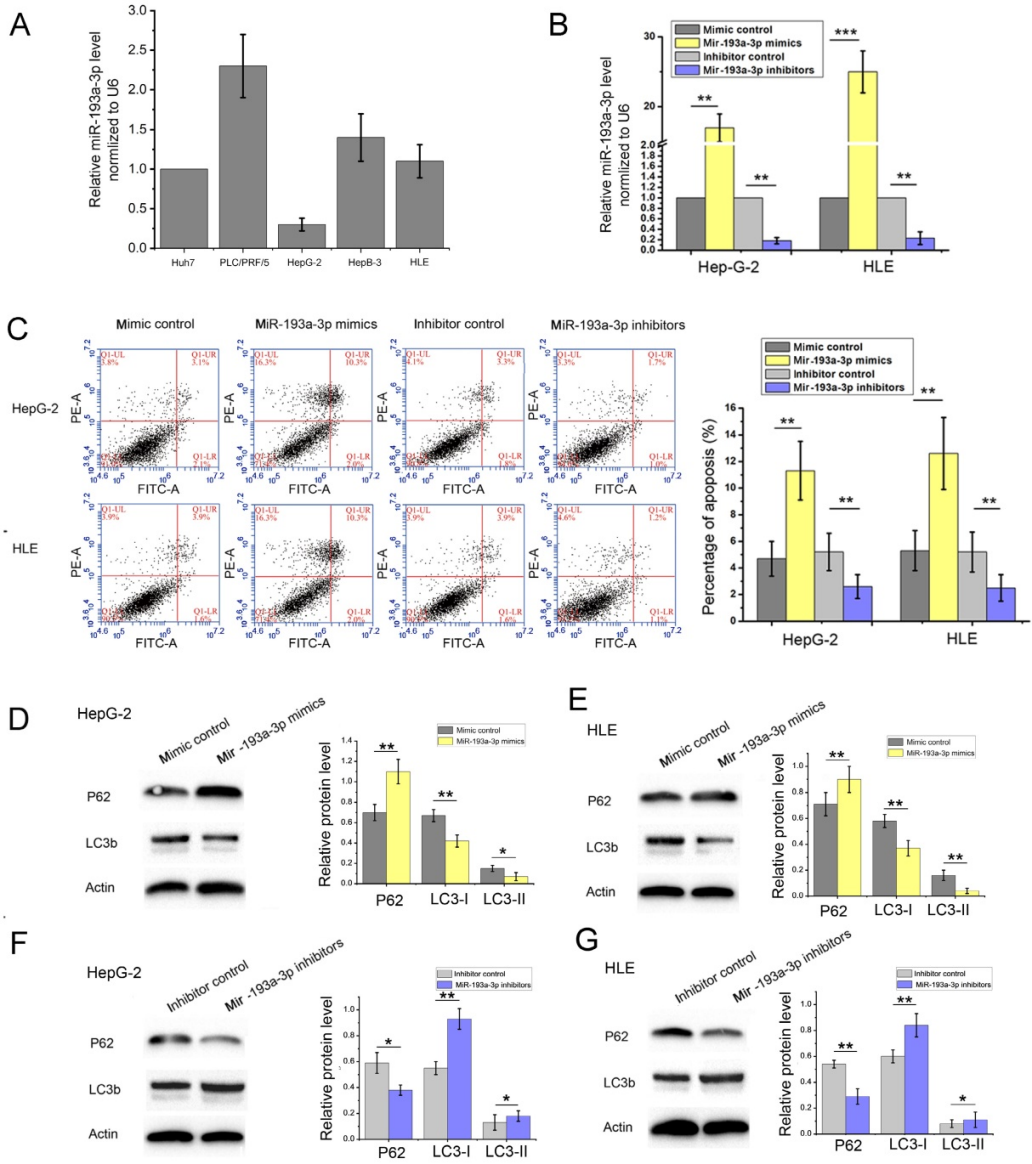
** Effect of miR-193a-3p on the apoptosis and autophagy-related proteins in HCC cell lines.** (A) Expression levels of miR-193a-3p in HCC cell lines. (B) Quantitative RT-PCR analysis of the expression levels of miR-193a-3p in HepG-2 and HLE cells transfected with miR-193a-3p mimics, inhibitors, and negative control. ^**^P < 0.01; ^***^P < 0.001. (C) Apoptosis assay 48 h after four groups of HepG-2 and HLE cells were transfected with equal doses of mimic control, miR-193a-3p mimics, inhibitor control, and miR-193a-3p inhibitors; the percentage of apoptotic cells was quantified. The cells in the Q1LR (Annexin V+/PI-) and Q1UR (Annexin V+/PI+) quadrants were recognized as apoptotic cells.^ **^P < 0.01. (D-G) Western blotting and quantitative analysis for Mig-6, LC3b, and p62 expression 48 h after transfection in HepG-2 and HLE cells in the aforementioned four groups. ^*^P < 0.05; ^**^P < 0.01.

miR-193a-3p is reported to be related to various pathological processes of liver cancer, including proliferation, migration, invasion, apoptosis, radioresistance, and chemotherapeutic tolerance. However, the relationship between miR-193a-3p and autophagy has not been reported to date. In this study, we first detected the expression levels of miR-193a-3p in HCC cell lines (Figure 4A). Then, we used real-time polymerase chain reaction (RT-PCR) to measure the transfection efficiency in HepG-2 and HLE cells (Figure 4B). We used miR-193a-3p mimics and miR-193a-3p inhibitors to study the function of miR-193a-3p. Mimics of miR-193a-3p were synthesized by chemical synthesis, which can enhance the function of endogenous miR-193a-3p. The miR-193a-3p inhibitor is a chemically modified inhibitor specifically targeting miR-193a-3p in cells.

**Figure 5:**
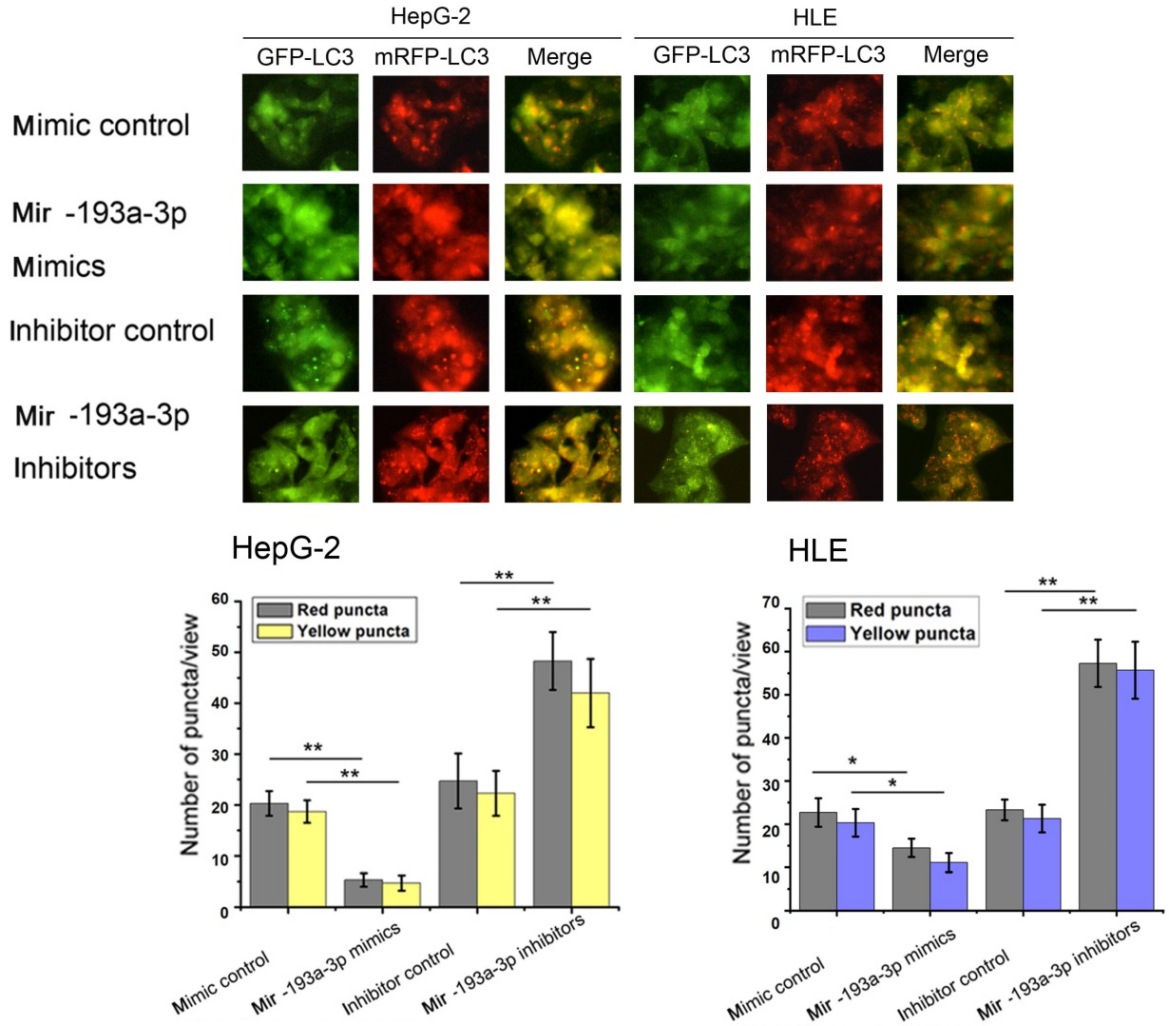
** Effect of miR-193a-3p on the autophagy of HCC cell lines.** Each group was transfected with a tandem mRFP-GFP-LC3 adenovirus for 24 h.Autophagosomes and autolysosomes were respectively visualized as yellow- and red-only punctas under a fluorescence microscope. ^*^P < 0.05; ^**^P < 0.01.

Then, apoptosis was inspected in HepG-2 and HLE cells using flow cytometric analysis. The percentage of apoptotic cells increased in cells transfected with miR-193a-3p mimics compared with the NC group; however, the cells transfected with miR-193a-3p inhibitors showed a decrease in the apoptotic rate (Figure 4C) Finally, the effect of miR-193a-3p on the autophagy capacity of HepG-2 and HLE cells was verified using western blot analysis and evaluation of the fluorescent LC3 experiment. The results showed that miR-193a-3p mimics inhibited autophagy, which was verified by the change in the expression of LC3b and p62 (Figure 4D-G), and significantly decreased the formation of autolysosomes (Figure 5). The reverse consequence was also observed in the miR-193a-3p inhibitor.

### miR-193a-3p inhibited autophagy of HCC by decreasing the expression of TGF-β2

**Figure 6:**
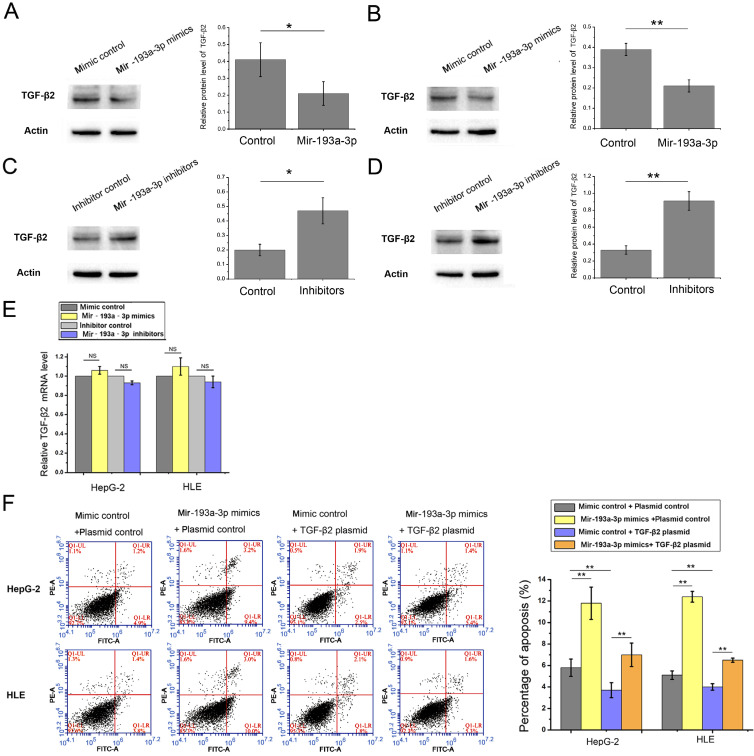
** Effect of miR-193a-3p and TGF-β2 plasmid on the apoptosis of HCC cell lines.** (A-D) Western blotting and the quantitative analysis of TGF-β2 protein levels in HepG-2 and HLE cells after transfection with equal doses of the miR-193a-3p mimics, inhibitors, and negative control. ^*^P < 0.05; ^**^P < 0.01. (E) Quantitative RT-PCR analysis of the expression levels of TGF-β2 mRNA in HepG-2 and HLE cells transfected with miR-193a-3p mimics, inhibitors, and negative control. (F) Apoptosis assay 48 h after four groups of HepG-2 and HLE cells were transfected with equal doses of mimic control plus plasmid control, miR-193a-3p mimics plus plasmid control, mimic control plus TGF-β2 plasmid, and miR-193a-3p mimics plus TGF-β2 plasmid. Cells in the Q1LR (Annexin V+/PI-) and Q1UR (Annexin V+/PI+) quadrants were recognized as apoptotic cells; the percentage of apoptosis cells was quantified.^ **^P < 0.01.

**Figure 7:**
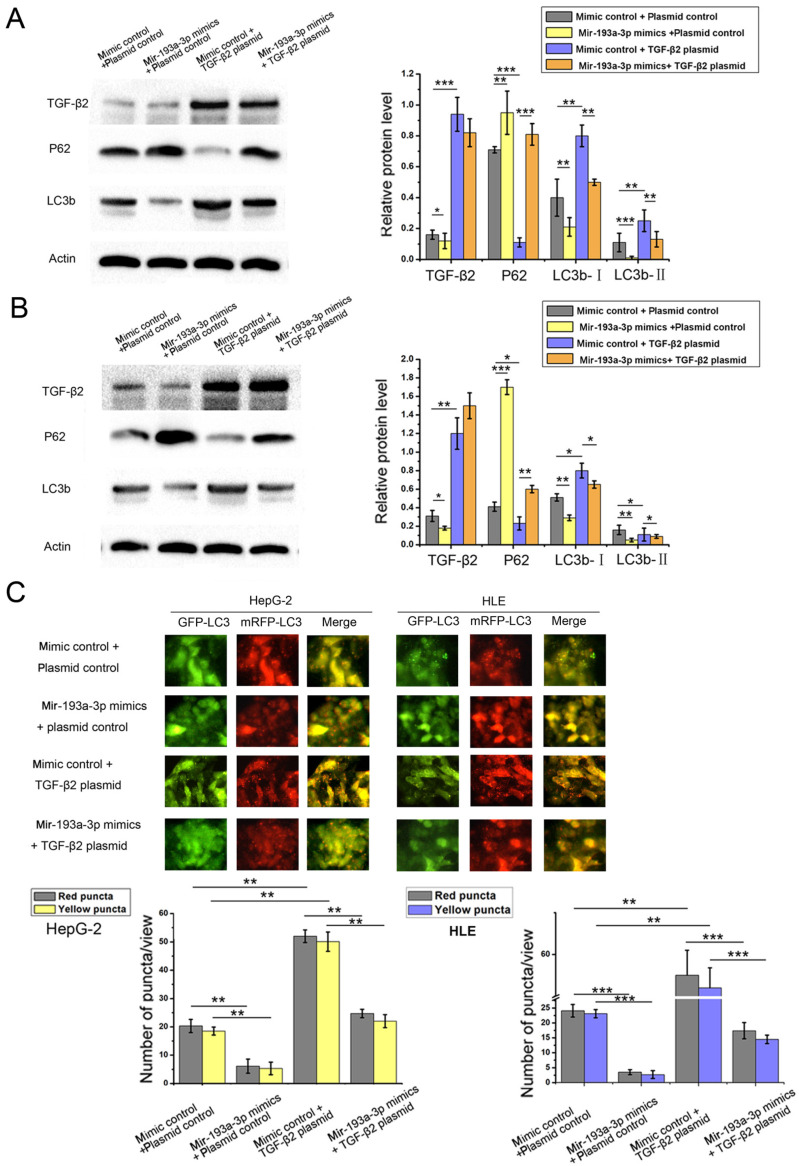
** Effect of miR-193a-3p and TGF-β2 plasmid on autophagy of the HCC cell lines.** (A and B) Western blotting and quantitative analysis for TGF-β2, LC3b, and p62 expression 48 h after the transfection in HepG-2 and HLE cells in the aforementioned four groups. ^*^P < 0.05; ^**^P < 0.01; ^***^P < 0.001. (C) Each group was transfected with a tandem mRFP-GFP-LC3 adenovirus for 24 h. Autophagosomes and autolysosomes were respectively visualized as yellow- and red-only punctas under a fluorescence microscope. ^**^P < 0.01; ^***^P < 0.001.

The miR-193a-3p target predicted by all three target gene prediction software programs was regarded with high confidence (Table S2). Thus, 62 nominated target genes were hit by miR-193a-3p. Among these, the TGF-β2 gene gained attention. Previous studies reported that TGF-β2 was regulated by multiple miRNAs and associated with apoptosis and autophagy in certain cell lines. In this study, the expression of TGF-β2 protein in both HepG-2 and HLE cells decreased after the transfection of miR-193a-3p mimics. The miR-193a-3p inhibitor significantly increased TGF-β2 protein expression (Figure 6A-D). After transfecting miR-193a-3p mimics or inhibitors into HCC cells, no obvious statistically significant difference was also found at the TGF-β2 mRNA level (Figure 6E). Next, the impact of miR-193a-3p and TGF-β2 on the apoptosis and autophagy of HCC cells was examined. The HepG-2 and HLE cells transfected with miR-193a-3p mimics showed an increased percentage of apoptotic cells and impaired the autophagy. However, TGF-β2 plasmid could not only inhibit apoptosis (Figure 6F) and enhance autophagy but also attenuated the decrease in autophagy caused by miR-193a-3p mimics (Figure 7A-C). These results substantiated that miR-193a-3p affected apoptosis and autophagy at least partly by controlling the expression of TGF-β2 in HCC cells.

### Mig-6 modulated apoptosis and autophagy of HCC cells through the miR-193a-3p and TGF-β2

**Figure 8:**
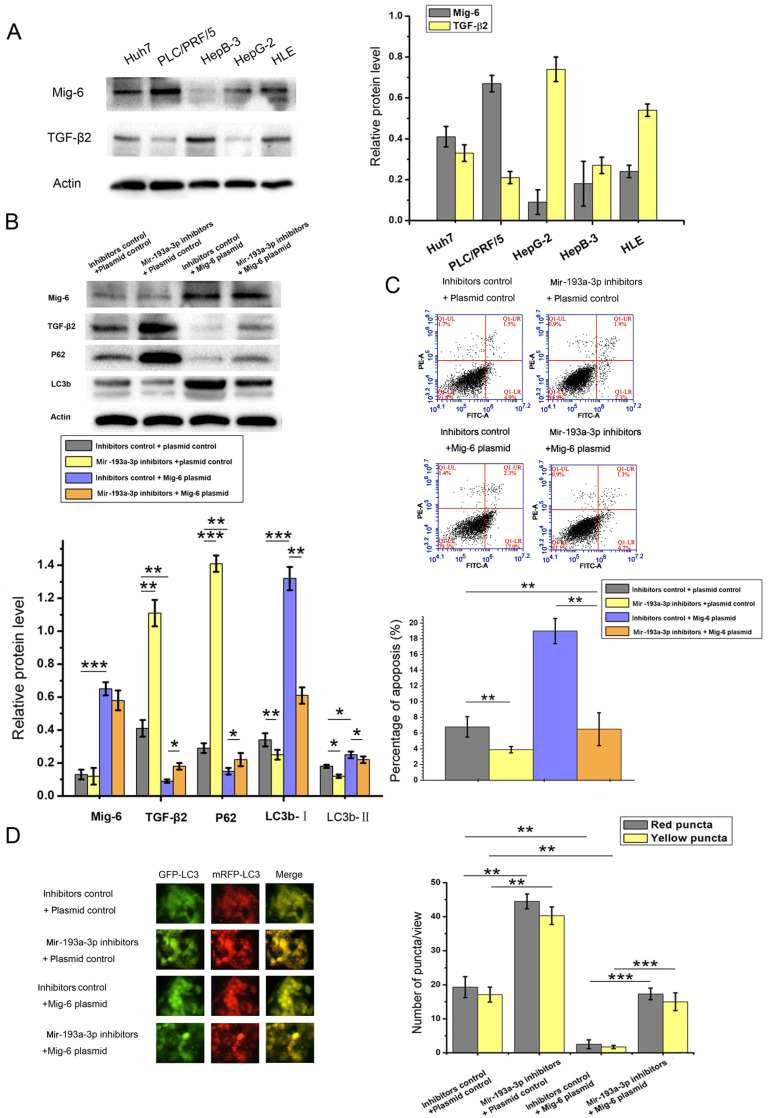
** Relationship between miR-193a-3p and TGF-β2 and the effect of Mig-6 plasmid and miR-193a-3p inhibitors on TGF-β2.** (A) Correlation of Mig-6 and TGF-β2in HCC cell lines. (B) Apoptosis assay 48 h after the transfection in HLE cells in the aforementioned four groups' transfection. Cells in the Q1LR (Annexin V+/PI-) and Q1UR (Annexin V+/PI+) quadrants were recognized as apoptotic cells.^ *^P < 0.05; ^**^P < 0.01; ^***^P < 0.001. (C) Western blotting and quantitative analysis for Mig-6, TGF-β2, LC3b, and p62 expression 48 h after the four groups of HLE cells were transfected with equal doses of inhibitor control plus plasmid control, miR-193a-3p inhibitors plus plasmid control, inhibitor control plus *Mig-6* plasmid, and miR-193a-3p inhibitors plus *Mig-6* plasmid. ^**^P < 0.01. (D) Each group was transfected with a tandem mRFP-GFP-LC3 adenovirus for 24 h. Autophagosomes and autolysosomes were respectively visualized as yellow- and red-only punctas under a fluorescence microscope. ^**^P < 0.01; ^***^P < 0.001.

The relationship between Mig-6 and TGF-β2 has not been reported to date. Therefore, the correlation between Mig-6 and TGF-β2 protein levels was analyzed in five HCC cell lines. As shown in Figure 6A, Mig-6 was highly expressed with low levels of TGF-β2 in Huh7 and PLC/PRF/5 cells. In HepB3 and HLE cells with the low expression of Mig-6, the expression of TGF-β2 was high. Therefore, an inverse relationship was observed between the expression of Mig-6 and TGF-β2 (Figure 8A). A series of function experiments were performed in HepG-2 and HLE cells to assess further impacts of the overexpression of Mig-6. As indicated by western blot analysis, the level of TGF-β2 protein decreased after HCC cells were transfected with Mig-6 plasmid, without any mRNA alteration (data not shown). Meanwhile, miR-193a-3p inhibitors reversed the decrease in TGF-β2 induced by the Mig-6 plasmid (Figure 8B). As shown in Figure 1A, the overexpression of Mig-6 led to a large percentage of apoptotic cells; this phenomenon could be rescued by miR-193a-3p inhibitors (Figure 8C). The autophagy levels of HCC cells were tested by western blot analysis and fluorescent LC3 puncta assay. The decreased autophagy due to the overexpression of Mig-6was reversed by miR-193a-3p inhibitors (Figure 8D).

## Discussion

Autophagy is a tightly regulated and highly conserved self-degradative process for balancing the sources of energy and in response to nutrient stress. Recent accumulating researches revealed a close relationship between autophagy and various human diseases, such as cancer 22. Apoptosis is widely known as programmed cell death. It is a highly regulated process by which cell death occurs in multicellular organisms. Apoptosis plays a crucial role in the pathogenesis of many cancer processes 23. Cross-talk between autophagy and apoptosis exists at different levels. In several cases, autophagy constitutes a stress adaptation that avoids cell death, whereas in other situations, autophagy can cause autophagic cell death. A good understanding of the relationship between autophagy and apoptosis would be important in present and future therapeutics for treating cancer and other diseases 24. This study elucidated that Mig-6 can inhibit the autophagy and then promote the apoptosis of HCC cells. Therefore, Mig-6 might exert its role by regulating the dynamic balance between apoptosis and autophagy in HCC cells.

As reported, Mig-6 can be regulated by certain miRNAs^25^-^28^. In particular, miR-589-5p and miR-374a regulate the proliferation of HCC cells by targeting Mig-6^29^, ^30^. These studies led to exploring whether Mig-6 could affect the changes in miRNAs. The miRNA microarray profiling was used to identify miRNAs specifically regulated by Mig-6. A total of 44 Mig-6-regulated miRNAs were identified, of which 11 were upregulated and 33 downregulated. Among these miRNAs, four miRNAs were upregulated more than 2.5 times by Mig-6, and miR-193a-3p was one of them.

A previous study indicated that miR-193a-3p was a tumor suppressor in many types of cancers 31. It inhibited the migration and invasiveness of NSCLC 32.In colorectal cancer, miR-193a-3p inhibited cell proliferation and promoted apoptosis 33. miR-193a-3p suppressed ovarian cancer cell growth and migratory and invasive capacities 34. miR-193a-3p had also been proven to be related to the migration and invasion 17, proliferation and apoptosis 18, 19, radioresistance, and chemotherapeutic tolerance in HCC^35^, ^36^. In addition, the expression of miR-193a-3p was identified as a predictor of HCC progression-free survival 37, 38. These results demonstrated the important role of miR-193a-3p in HCC. In this study, miR-193a-3p mimics induced a high level of apoptosis in HCC cell lines; however, miR-193a-3p inhibitors decreased the apoptotic rate, which was consistent with previous findings. Also, the autophagic capacity of HCC cell lines was found to be reduced by miR-193a-3p mimics, whereas miR-193a-3p inhibitor could enhance autophagy. Taken together, these results indicate that miR-193a-3p exerts its anti-cancer effect by influencing cell autophagy and apoptosis in HCC.

miR-193a-3p has been reported to have multiple target genes^32^, ^34^, ^39^, ^40^.In this study, TargetScan, miRNA.ORG, and miRDB software programs were used for predicting target genes. Finally, TGF-β2 was selected, which might play an important role in HCC as the target of miR-193a-3p. TGF-β has three different isoforms: TGF-β1, TGF-β2, and TGF-β3, with similar but not identical biologic activities 41. The role of TGF-β signaling in HCC progression is not completely understood. Tumor cells could escape from immune surveillance by TGF-β2 induced immunosuppression 42. TGF-β2 was also thought to be a promising therapeutic target in HCC owing to its overexpression 43. Exogenous TGF-β2 resulted in a significant elevation of the epithelial-to-mesenchymal transition by enhancing autophagy in HCC 44. This study eventually concluded that the upregulation of TGF-β2 could inhibit apoptosis and promote the autophagy of HCC cells. It also confirmed the inverse correlation between Mig-6 and TGF-β2.

This study had some limitations that are worth mentioning. First, the clinical significance of the interaction among Mig-6, miR-193a-3p, and TGF-β2 in HCC needed to be analyzed, which could increase the value of Mig-6, miR-193a-3p, and TGF-β2 in the diagnosis. Second, the overexpression of Mig-6 or Mig-6 gene knockout mice could be applied to verify the relationship between Mig-6, miR-193a-3p, and TGF-β2 *in vivo*. Third, although the direct binding of miR-193a-3p and TGF-β2 was detected in the LX2 cell line 45 , we did not perform this experiment in the present study. Further experiments need to be carried out in the liver cancer cell line. Fourth, many miRNAs regulated by Mig-6 were also found in this study, but their mechanism was not studied. Subsequent experiments need to explore the new functions of Mig-6-regulated miRNAs in HCC.

Specific challenges remain to our understanding of Mig-6 as the tumor suppressors in HCC. In this study, our data verified that Mig-6 and miR-193a-3p could promote the apoptosis and inhibition autophagy by regulating the expression of targeting TGF-β2. The Mig-6 regulated miRNAs and the correlation between Mig-6 and TGF-β2 were also confirmed in HCC for the first time. A study on Mig-6, miR-193a-3p, and TGF-β2 will provide more perspectives on the treatment of HCC.

## Supplementary Material

Supplementary tables.

## Figures and Tables

**Table 1:** Expression changes of Mig6-regulated miRNAs

miRNA	Regulation	Control virus or LV-Mig-6 Fold change
hsa-miR-1269a	Up	3.663214041
hsa-miR-193a-3p	Up	2.960149418
hsa-miR-1244	Up	2.512778203
hsa-miR-371b-5p	Up	2.503430956
hsa-miR-5093	Down	-5.363445158
hsa-miR-297	Down	-4.571560354
hsa-miR-4793-3p	Down	-4.556744037
hsa-miR-610	Down	-4.539741026
hsa-miR-4440	Down	-4.535851264
hsa-miR-195-3p	Down	-4.458473988
hsa-miR-6877-3p	Down	-4.173369522
hsa-miR-3148	Down	-3.956889197
hsa-miR-8071	Down	-3.647623099
hsa-miR-185-3p	Down	-3.48629987
hsa-miR-3064-5p	Down	-3.347459653
hsa-miR-595	Down	-3.344313283
hsa-miR-3907	Down	-3.319532324
hsa-miR-7844-5p	Down	-2.856073948
hsa-miR-4455	Down	-2.736743073
hsa-mir-297	Down	-2.606974853
