# Supplementary material for: Mig-6 Inhibits Autophagy in HCC Cell Lines by Modulating miR-193a-3p
Source: Int J Med Sci. 2022 Jan 16;19(2):338–51. doi: 10.7150/ijms.66040 (PMC8795807; doi:10.7150/ijms.66040)
Supplement: Supplementary file 1 — Supplementary tables. [file ijmsv19p0338s1.pdf]

Supplement Table 1 Primer information

Name	Code
hsa-miR-1269a Primer	MQPS0000530
hsa-miR-193a-3p Primer	MQPS0000750-
hsa-miR-1244 Primer	MQPS0000488
hsa-miR-297 Primer	MQPS0000901
hsa-miR-4793-3p Primer	MQPS0001614
hsa-miR-610 Primer	MQPS0001965
U6 Primer	MQPS0000002

Supplement Table 2 The target genes of miR193a-3p

Symbol	TargetScan	microRNA.ORG	miRDB	Count
FAT4	Y	Y	Y	3
EN2	Y	Y	Y	3
KMT2A	Y	Y	Y	3
ARHGEF12	Y	Y	Y	3
YWHAZ	Y	Y	Y	3
TGFB2	Y	Y	Y	3
TMEM30A	Y	Y	Y	3
PAK4	Y	Y	Y	3
MSANTD2	Y	Y	Y	3
SOS2	Y	Y	Y	3
ETV1	Y	Y	Y	3
SLC10A6	Y	Y	Y	3
LAMC1	Y	Y	Y	3
KIT	Y	Y	Y	3
CNBP	Y	Y	Y	3

ERBIN	Y	Y	Y	3
FAM168B	Y	Y	Y	3
CADM1	Y	Y	Y	3
SLC16A6	Y	Y	Y	3
FAM84A	Y	Y	Y	3
NOVA1	Y	Y	Y	3
LAMP2	Y	Y	Y	3
ERBB4	Y	Y	Y	3
AJUBA	Y	Y	Y	3
TAOK1	Y	Y	Y	3
DAAM2	Y	Y	Y	3
CAPRIN1	Y	Y	Y	3
GNAO1	Y	Y	Y	3
SELENON	Y	Y	Y	3
ADAMTSL3	Y	Y	Y	3
ETV6	Y	Y	Y	3
DUSP7	Y	Y	Y	3
LRP4	Y	Y	Y	3
SLC39A5	Y	Y	Y	3
TNFRSF21	Y	Y	Y	3
FLI1	Y	Y	Y	3
ARMC1	Y	Y	Y	3

TBL1XR1	Y	Y	Y	3
MCL1	Y	Y	Y	3
ZBTB5	Y	Y	Y	3
BRPF1	Y	Y	Y	3
TSC1	Y	Y	Y	3
FLRT3	Y	Y	Y	3
SCYL3	Y	Y	Y	3
UBP1	Y	Y	Y	3
INO80D	Y	Y	Y	3
TCF4	Y	Y	Y	3
SRSF2	Y	Y	Y	3
STMN1	Y	Y	Y	3
KIAA1549L	Y	Y	Y	3
HOXD13	Y	Y	Y	3
ING5	Y	Y	Y	3
WDR82	Y	Y	Y	3
STX16	Y	Y	Y	3
TP53INP1	Y	Y	Y	3
NT5E	Y	Y	Y	3
GRB7	Y	Y	Y	3
IRF2BPL	Y	Y	Y	3
ANAPC15	Y	Y	Y	3

SRSF6	Y	Y	Y	3
JADE2	Y	Y	Y	3
ST6GALNAC5	Y	Y	Y	3
NRIP1	Y	Y	Y	3
ZNF248	Y	Y	Y	3
RUNX1T1	Y	Y	Y	3
LAMC2	Y	Y	Y	3
IL17RD	Y	Y	Y	3
SNX27	Y	Y	Y	3
CALB1	Y	Y	Y	3
KRAS	Y	Y	Y	3
JMY	Y	Y	Y	3
ZNF385B	Y	Y	Y	3
TGFBR3	Y	Y	Y	3
CBX7	Y	Y	Y	3
MYCN	Y	Y	Y	3
NSF	Y	Y	Y	3
DYRK2	Y	Y	Y	3
HEG1	Y	Y	Y	3
ING1	Y	Y	Y	3
CSRNP1	Y	Y	Y	3
GDF11	Y	Y	Y	3

DNAJC13	Y	Y	Y	3
ZC3H11A	Y	Y	Y	3
FHDC1	Y	Y	Y	3
CNOT6	Y	Y	Y	3
AP2M1	Y	Y	Y	3
PLAU	Y	Y	Y	3
RSF1	Y	Y	Y	3
MMP19	Y	Y	Y	3
CCND1	Y	Y	Y	3
CTDSPL2	Y	Y	Y	3
